# Supplementary material for: Characterizing cell-type spatial relationships across length scales in spatially resolved omics data
Source: Nat Commun. 2025 Jan 3;16:350. doi: 10.1038/s41467-024-55700-1 (PMC11699133; doi:10.1038/s41467-024-55700-1)
Supplement: Supplementary file 3 — Supplementary Software 1 [file 41467_2024_55700_MOESM3_ESM.zip › CRAWDAD/plots/supplementary/S7_spleen_tsne_datasets_separate_beforeHarmonyTsne.pdf]

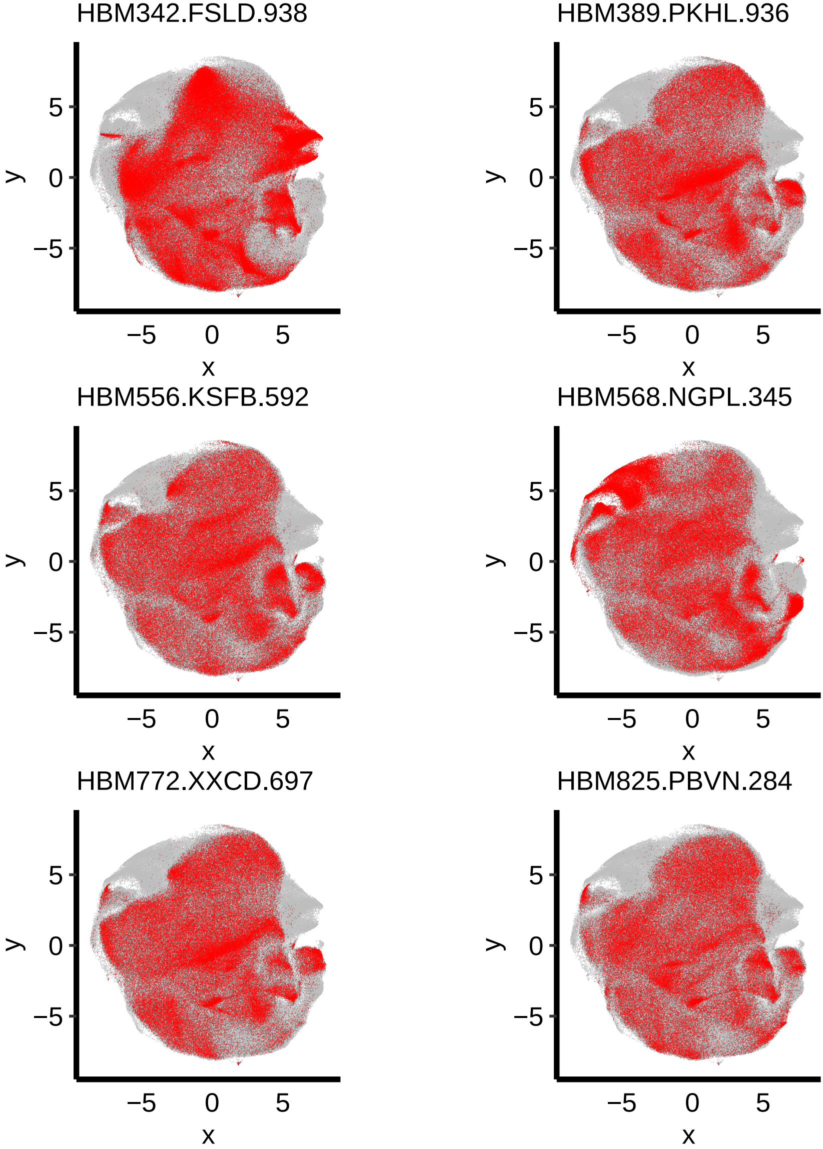
HBM342.FSLD.938
HBM389.PKHL.936
HBM556.KSFB.592
HBM568.NGPL.345
HBM772.XXCD.697
HBM825.PBVN.284
y
x
5
0
−5
